# A rare cause of portal vein thrombosis: Fascioliasis

**DOI:** 10.1590/0037-8682-0026-2026

**Published:** 2026-04-17

**Authors:** Sonay Aydin, Zulaika Askarbek Kyzy, Kemal Bugra Memis

**Affiliations:** 1 Erzincan Binali Yıldırım University, Faculty of Medicine, Department of Radiology, Erzincan 24100, Turkey.

A 12-year-old girl presented to the pediatric clinic with a one-month history of abdominal and chest pain. Systemic examination revealed no significant abnormalities except for mild right upper quadrant tenderness. Laboratory tests showed severe eosinophilia. Abdominal ultrasonography revealed a lobulated hypoechoic lesion measuring approximately 3 × 2 cm in segment VI of the liver (Figure 1). Dynamic contrast-enhanced magnetic resonance imaging of the liver revealed a 2-cm tubular lesion with contrast enhancement along the bile ducts in segment VII of the liver. A perfusion defect was also observed due to thrombosis of the portal vein branch within this segment (Figure 2 and Figure 3). These imaging findings suggested infection with *Fasciola hepatica*, which was subsequently confirmed by serological testing. Treatment with triclabendazole (10 mg/kg) was initiated. 

**FIGURE 1:**
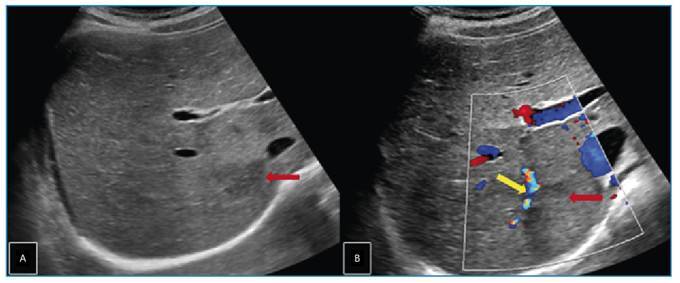
Transverse gray scale **(A)** and color Doppler **(B)** ultrasonography images of the liver showing an ill-defined hypoechoic lesion **(red arrows)** in segment VI and focal thrombosis **(yellow arrow)** in the adjacent portal vein branch.

**FIGURE 2:**
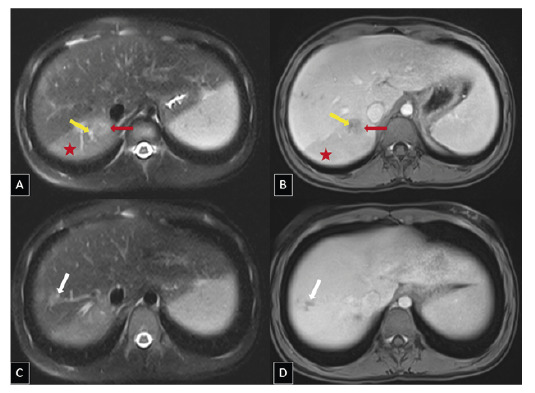
Axial T2-weighted fat-suppressed **(A, C)** and axial T1-weighted fat-suppressed post-contrast venous phase **(B, D)** magnetic resonance images showing a poorly defined T2-hyperintense parenchymal parasitic lesion in segment VI **(red arrows)** with minimal contrast enhancement. Focal thrombosis is seen in the portal vein branch supplying the posterior regions of the right lobe **(yellow arrows)**, with associated perfusion alterations in the parenchyma **(red asterisks)**. Localized bile duct dilation is also noted in segment VII **(white arrows)**, indicating its potential association with the ductal phase.

**FIGURE 3:**
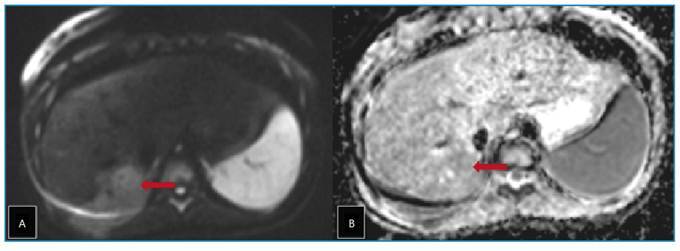
Axial diffusion-weighted magnetic resonance image **(A)** and corresponding apparent diffusion coefficient map **(B)** showing diffusion restriction within the parenchymal parasitic lesion **(red arrows)** in segment VI of the liver.

*Fasciola hepatica*, commonly known as the "liver fluke," is a trematode parasite that infests the bile ducts of mammals, including cattle, sheep, and humans^1^. Fascioliasis is a zoonotic infection acquired through ingestion of contaminated water or aquatic vegetation, such as watercress^2^. During pathogenesis, larvae migrate from the intestinal tract to the liver and infiltrate the bile ducts, causing inflammation and obstruction^1^ ^,^ ^2^. Hepatomegaly, biliary obstruction, secondary bacterial infections, and portal vein thrombosis may occur^3^. This case underscores the importance of considering fascioliasis in paediatric patients with persistent abdominal pain. Eosinophilia and specific imaging findings facilitate early and accurate diagnosis. Early detection may prevent irreversible biliary damage and serious complications, including portal vein thrombosis^3^ ^,^ ^4^. 

## References

[1] Dusak A, Onur MR, Cicek M, Firat U, Ren T, Dogra VS (2012). Radiological Imaging Features of Fasciola hepatica Infection - A Pictorial Review. J Clin Imaging Sci.

[2] Pritsch IC, Garcia RL, Douat D, Schwendler RR, Buttendorf MRB, Molento MB (2019). First reported case of clinical fascioliasis in Santa Catarina, Brazil. Rev Soc Bras Med Trop.

[3] Alave J, Leon M, Terashima A, Concha-Velasco F, Gotuzzo E, Seas C (2024). Subcapsular Liver Hematoma: One of the Many Faces of Acute Fascioliasis. Open Forum Infect Dis.

[4] Girma A, Belete Y, Afework S, Bisrat T (2024). The Liver's hidden foe: A case study on human fasciolasis. IDCases.

